# Supplementary figures and images for: Random forest vs. logistic regression: Predicting angiographic in-stent restenosis after second-generation drug-eluting stent implantation
Source: PLoS One. 2022 May 23;17(5):e0268757. doi: 10.1371/journal.pone.0268757 (PMC9126385; doi:10.1371/journal.pone.0268757)

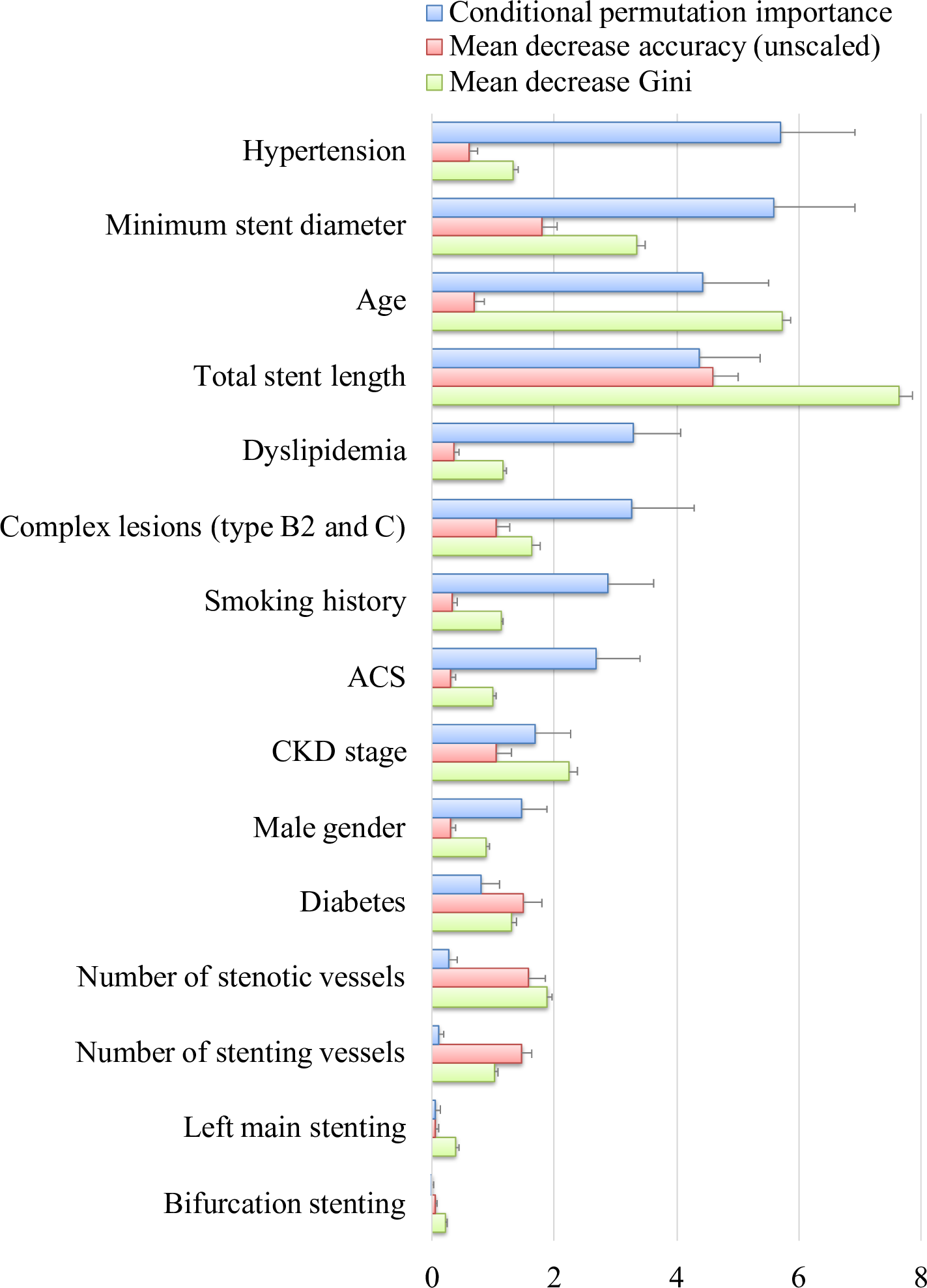

Supplement: S1 Fig — The conditional permutation importance, mean decrease accuracy, and mean decrease Gini of the 15 variables. Order as descending CPI value. The higher the value, the more important the variable is. Abbreviations: ACS = acute coronary syndrome, CKD = chronic kidney disease. (TIF) [file pone.0268757.s001.tif]

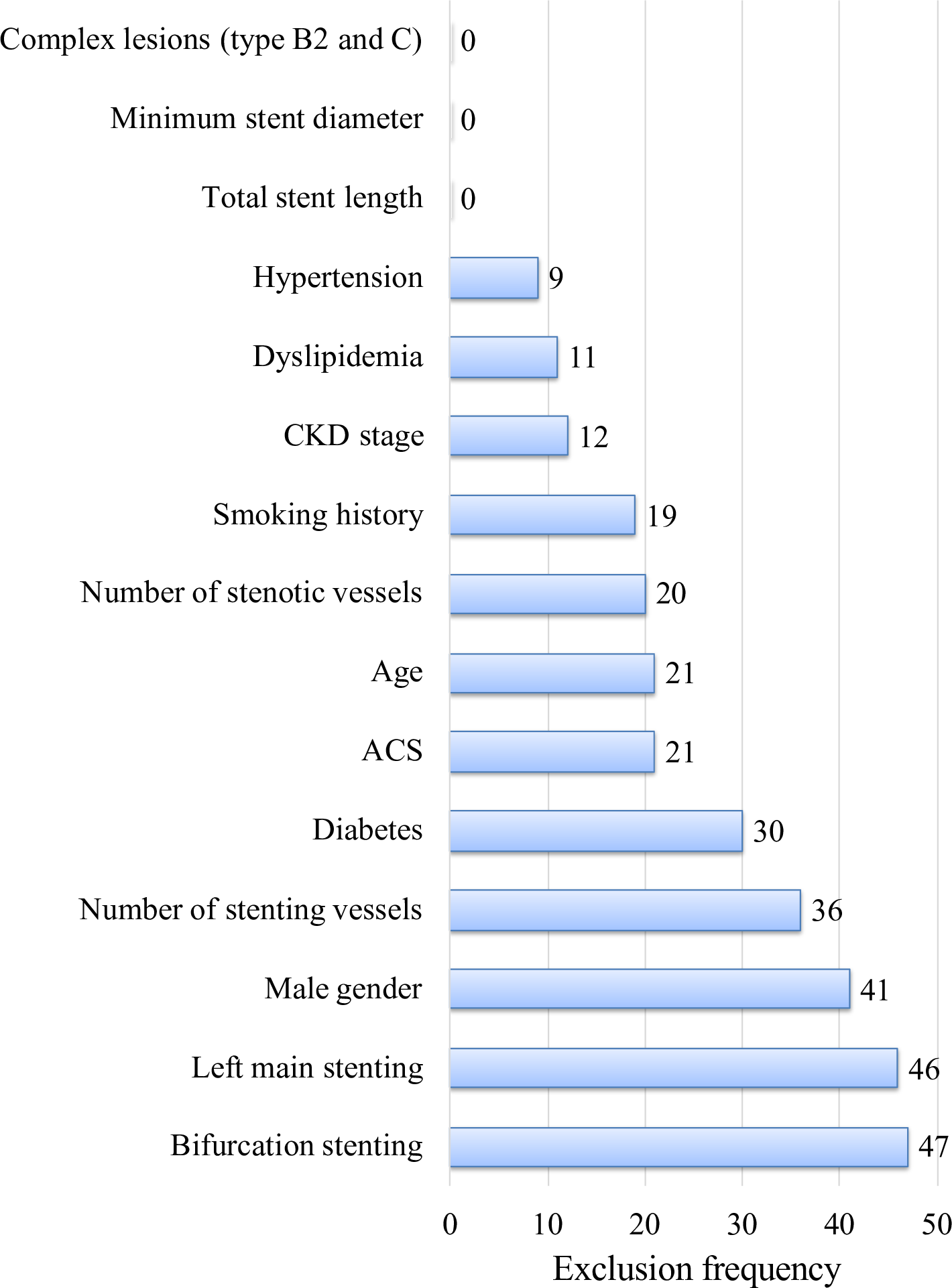

Supplement: S2 Fig — The exclusion frequency was counted from the 50 LR models. The exclusion frequency is denoted at the top of each column. Order as ascending exclusion frequency. The higher the frequency, the variable less influenced the LR model. Abbreviations: ACS = acute coronary syndrome, CKD = chronic kidney disease. (TIF) [file pone.0268757.s002.tif]

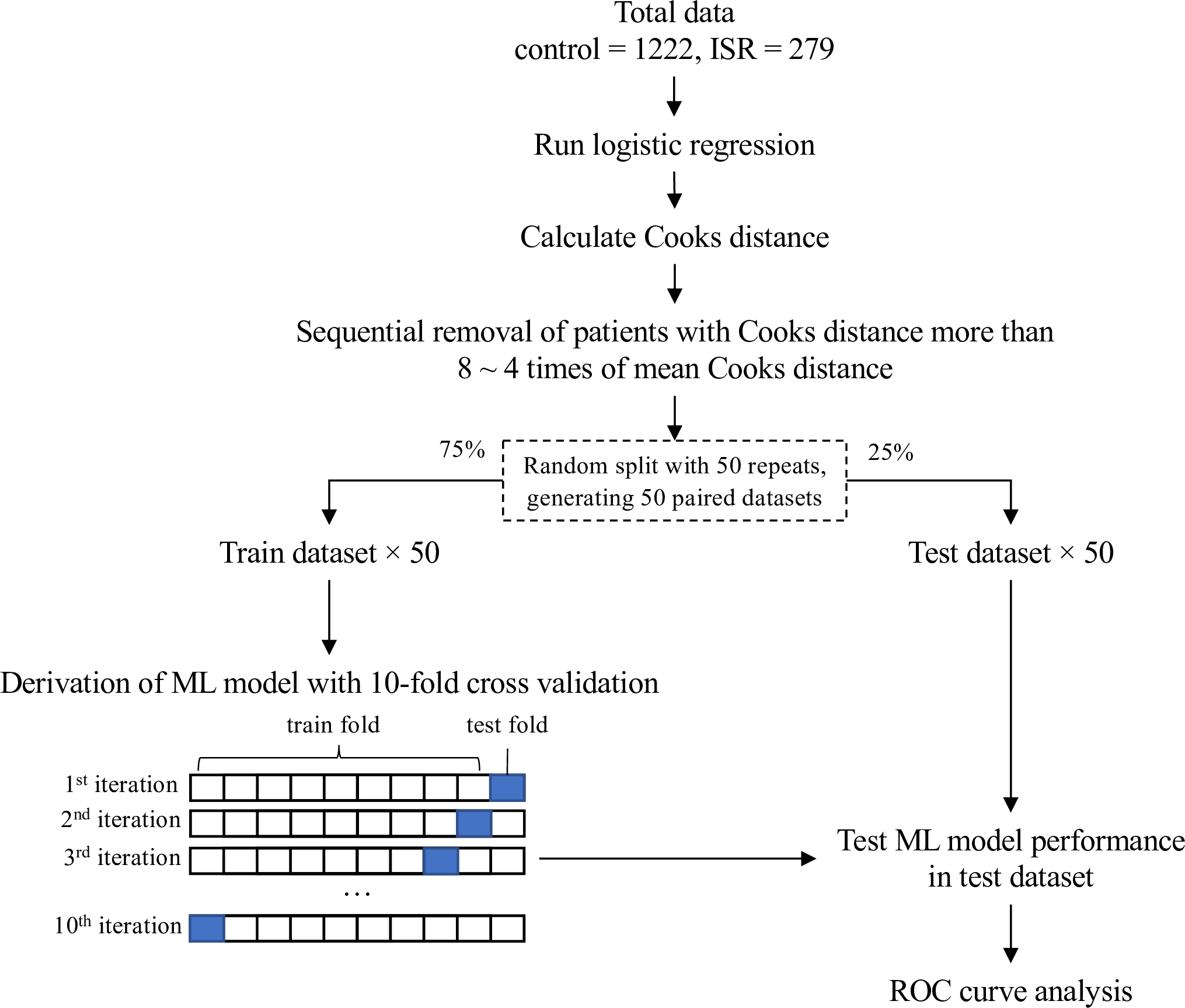

Supplement: S3 Fig — Abbreviations: ISR = intra-stent restenosis, ROC = receiver operating characteristic. (TIF) [file pone.0268757.s003.tif]

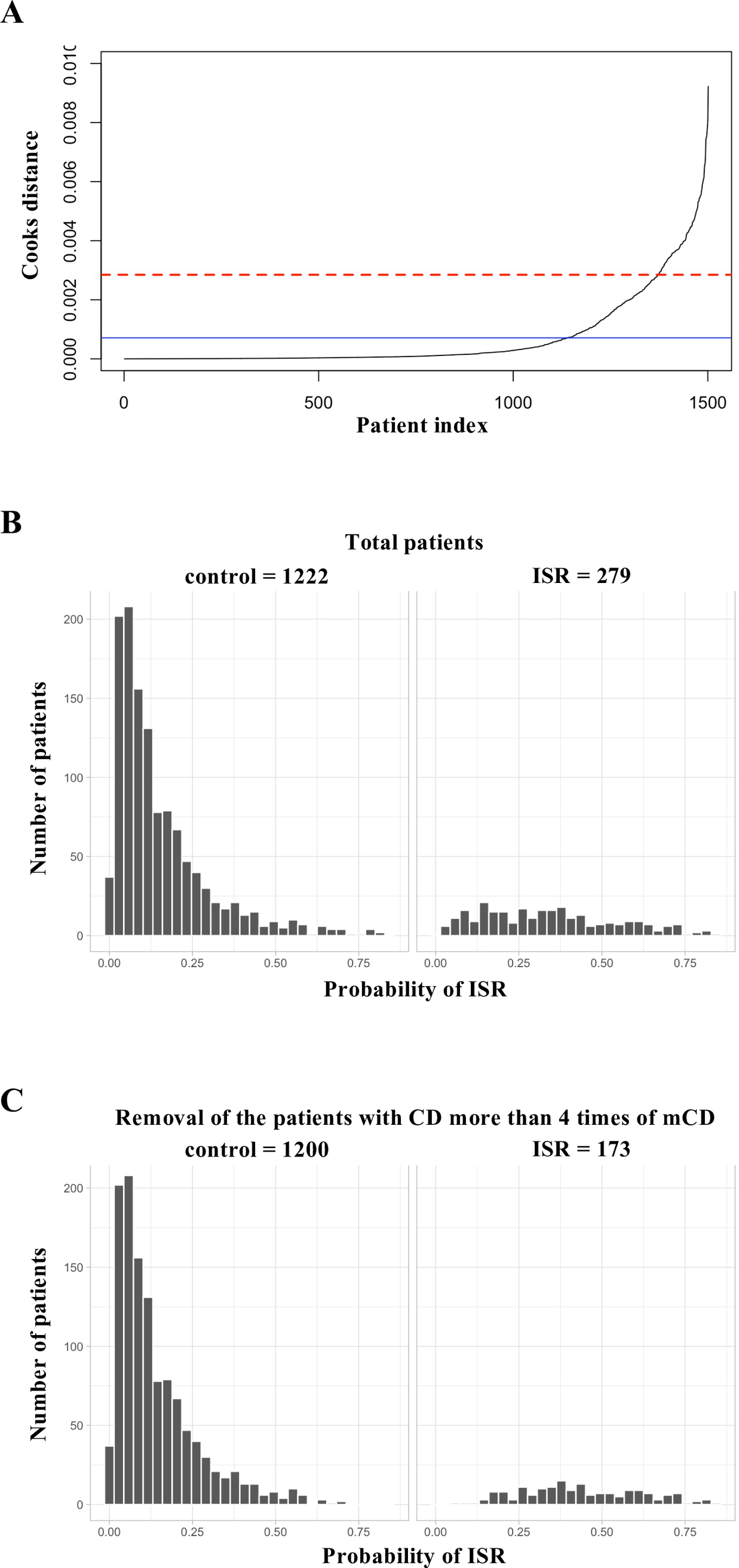

Supplement: S4 Fig — The Cooks distances among the study population (A). The X-axis denotes each patient. The Y-axis denotes the Cooks distance of each patient in ascending order. The blue solid line denotes the mCD. The red dashed line denotes the threshold of 4 times of mCD. The histogram of the probability of ISR of the study population (B) and that after removal of the patients with more than 4 times of mCD (C). The X-axis ranges from 0 to 1, denoting the probability of ISR. The Y-axis denotes the patient frequency of the probabilities. Abbreviations: ISR = in-stent restenosis; mCD = mean Cooks distance. (TIF) [file pone.0268757.s004.tif]

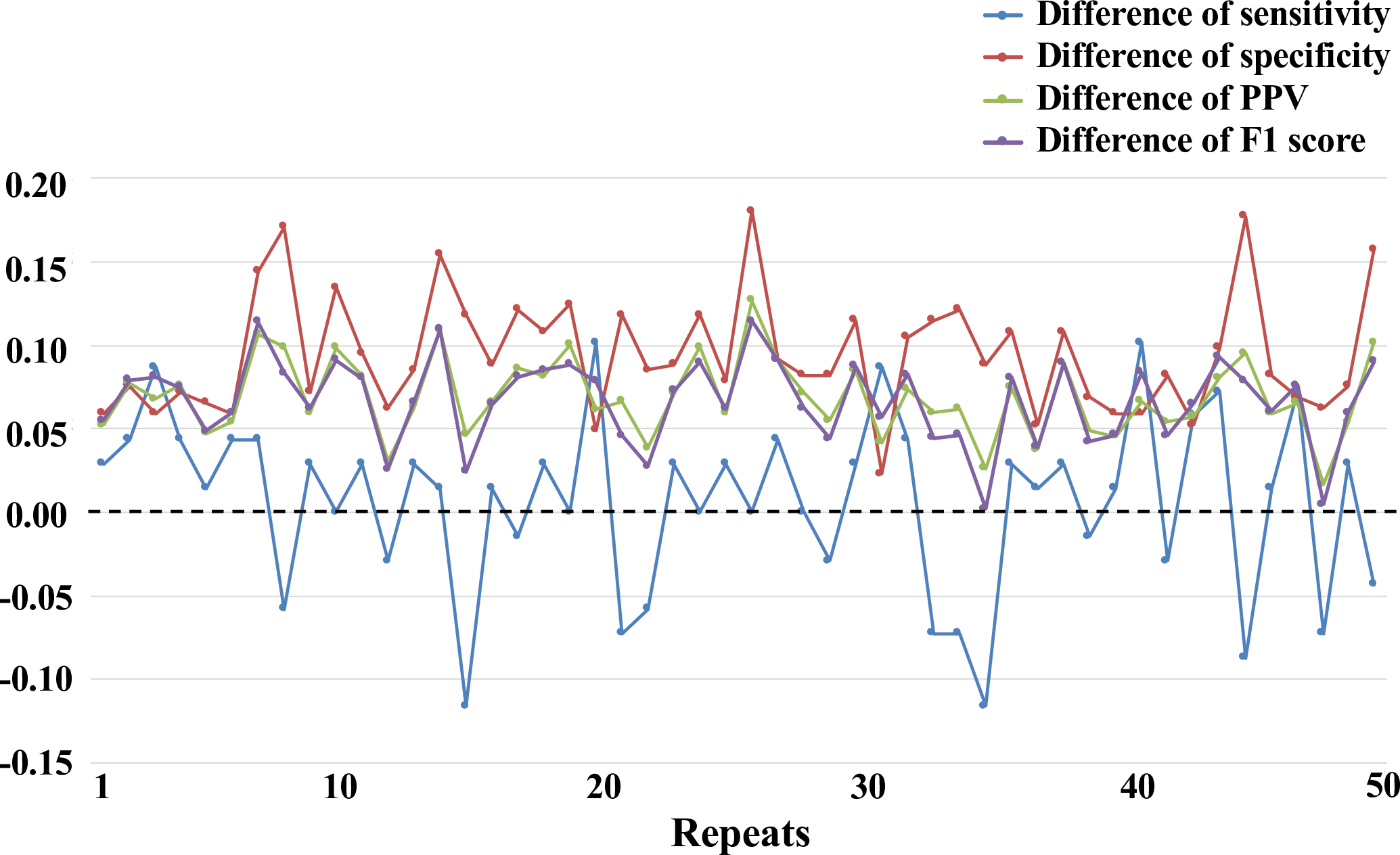

Supplement: S5 Fig — The difference was calculated by subtracting the value of the LR model from that of the RF model in each test dataset. The difference of sensitivity oscillated around zero in the 50 test datasets. However, the differences in specificity, PPV, and F1 scores were all above zero, indicating that the RF models had higher specificity, PPV, and F1 scores than the LR models under similar sensitivity. Abbreviations: LR = logistic regression; RF = random forest. (TIF) [file pone.0268757.s005.tif]
